# Prophylactic Efficacy of Melatonin on Cyclophosphamide-Induced Liver Toxicity in Mice

**DOI:** 10.1155/2014/470425

**Published:** 2014-06-30

**Authors:** Mohammad Shokrzadeh, Amirhossein Ahmadi, Farshad Naghshvar, Aroona Chabra, Mehdi Jafarinejhad

**Affiliations:** ^1^Pharmaceutical Sciences Research Center, Faculty of Pharmacy, Mazandaran University of Medical Sciences, 18 Kilometers of Farah Abad Road, Sari 48175-861, Iran; ^2^Department of Toxicology and Pharmacology, Faculty of Pharmacy, Mazandaran University of Medical Sciences, 18 Kilometers of Farah Abad Road, Sari 48175-861, Iran; ^3^Department of Pathology, Faculty of Medicine, Mazandaran University of Medical Sciences, 18 Kilometers of Farah Abad Road, Sari 48175-861, Iran; ^4^Student Research Committee, Mazandaran University of Medical Sciences, 18 Kilometers of Farah Abad Road, Sari 48175-861, Iran

## Abstract

The current study aimed to evaluate the protective effects of melatonin, a pineal secretory product, against hepatotoxicity induced by cyclophosphamide (CP) in mice. Mice were pretreated with melatonin intraperitoneally for 7 consecutive days before the administration of a single intraperitoneal dose of 200 mg/kg CP. 24 hr after CP administration, the mice were anesthetized, blood was then removed, and serum toxicity enzymes activities were evaluated. After the blood sampling, all animals were killed, livers were then removed, and histological studies were conducted. Serum toxicity marker enzymes were significantly increased after CP treatment but restored in melatonin pretreated groups. In addition, administration of CP induced necrotic hepatocyte with small crushed nuclei, portal space with severe inflammation, and hepatocytes surrounded by lymphocytic infiltration in hepatic tissues. However, melatonin effectively protected against CP-induced histopathological abnormalities in the liver tissues. Our results reveal that melatonin produces a potent hepatoprotective mechanism against CP. Therefore, melatonin could be a potent candidate to use concomitantly as a supplement agent against hepatotoxicity of CP for the patients undergoing chemotherapy.

## 1. Introduction

Cyclophosphamide (N,N-bis(2-chloroethyl)tetrahydro-2H-1,3,2-oxazaphosphorin-2-amine 2-oxide, CP), an oxazaphosphorine derivative of the classical alkylating agent nitrogen mustard, is commonly used in cancer chemotherapy. This drug also has significant immunosuppressive activity and is used clinically in the treatment of autoimmune diseases and for renal and bone marrow transplantations [1]. However, despite its wide spectrum of clinical uses, CP also possesses a wide spectrum of adverse effects including hepatotoxicity in humans and experimental animals [2, 3]. The precise mechanism by which CP causes hepatic injury is poorly known. However, CP requires metabolic activation by hepatic microsomal cytochrome P450 mixed function oxidase system for both its therapeutic action and its toxicologic actions [1]. Metabolic conversion of CP leads to the formation of two cytotoxic metabolites: phosphoramide mustard and acrolein. Phosphoramide mustard is believed to have an antineoplastic activity, while acrolein, a highly reactive metabolite with a short biological half-life, may be responsible for CP-induced liver injury [4]. Recent studies suggest that CP generates reactive oxygen species (ROS) like superoxide anion, hydroxyl radical, and hydrogen peroxide (H_2_O_2_) during its oxidative metabolism and depresses the antioxidant defense mechanisms in the liver [5, 6]. Regarding the above changes in the cells induced by CP, it is significant to find a compound capable of protecting the healthy cells and tissues against the activity of CP metabolites such as acrolein and free radicals. Various studies show that antioxidant intake can control the reaction to chemotherapy and also minimize the adverse side effects of antineoplastic drugs [7].

Melatonin (N-acetyl-5-methoxytryptamine) is synthesized mainly by the pineal gland and is suggested to have antioxidant and protective effects against oxidative stress in several experimental and clinical conditions [8]. Melatonin possesses strong antioxidant activity by which it protects cells, tissues, and organs from the oxidative damage caused by reactive oxygen species, especially the hydroxyl radical (^*∙*^OH), which attacks DNA, proteins, and lipids and causes pathogenesis [9]. Melatonin can quench the peroxyl radical, hypochlorous acid, and singlet oxygen, all of which cause cell damage [10–12]. The direct effects of melatonin on the male reproductive system and testosterone synthesis from Leydig cells have also been examined in studies on animals [13]. Melatonin also prevents oxidative damage of the liver induced by ischemia reperfusion [14]. In our recent study, melatonin was shown to reduce the testicular toxicity induced by CP in male mice through antioxidant activity and free radical-scavenging properties [15]. We also showed that melatonin had a potent chemoprotective effect against the genotoxicity induced by diazinon in human blood lymphocyte cells, and this protective effect might be the result of free radical-scavenging properties [16].

Because melatonin has excellent antioxidative properties, there is a likely possibility that melatonin would protect against the toxicity of cyclophosphamide; that is, an elevated level of melatonin in body may act as a prophylactic against hepatic damage. Thus, the aim of this study was to determine if melatonin has hepatoprotective effects. Histological examination of liver tissues was also used to determine any morphological influence of melatonin on CP toxicity.

## 2. Materials and Methods

### 2.1. Animals

Male Naval Medical Research Institute (NMRI) mice weighing 28 ± 4 g were obtained from the Pasteur Institute of Iran (Amol), kept in animals' house of the university animal facility, and maintained under a controlled 12 hr light/dark cycle and temperature (24 ± 1°C). The animals were acclimatized for 1 week before the study and were given standard food pellets and water* ad libitum*. All procedures were performed according to the “Care and Use of Laboratory Animals” prepared by the Mazandaran University of Medical Sciences, Sari, Iran. The protocol for the study was approved by the Research Committee of the University.

### 2.2. Experimental Treatment

For the experiment, animals were divided into six groups each of five mice as follows.

In negative control, mice received distilled water (10 mL/kg b.w.) via intraperitoneal (i.p.) injection for 7 days. In positive control, mice received a single toxic dose of CP (200 mg/kg b.w., i.p.) in distilled water (10 mL/kg b.w.). In groups 3–6, mice were treated with melatonin at different concentrations (2.5, 5, 10, and 20 mg/kg b.w. by i.p. injection) in distilled water (10 mL/kg b.w.) per day for 7 consecutive days followed by a single i.p. dose of CP 1 hr after the last dose of melatonin. 24 hrs after CP administration, the mice were anesthetized with petroleum ether. 2 mL of blood was then removed with a cardiac puncture, and serum was separated and rapidly frozen at−80°C for later analysis to determine the serum toxicity enzymes activities. After the blood sampling, all animals were killed by an overdose of ether. The livers were then removed and washed three times by normal saline for complete blood removal. The livers were used for histopathological examinations.

### 2.3. Estimations of Serum Toxicity Marker Enzymes Activities

The extent of hepatic damage is assessed by the levels of released cytoplasmic alkaline phosphatase (ALP) and transaminases, including alanine transaminase (ALT) and aspartate transaminase (AST), in circulation [17]. Serum toxicity marker enzymes activities, ALT, AST, and ALP, were evaluated based on our previous experiment [18]. For the estimation of ALT and AST activities in serum samples, commercially available enzymatic kits (based on the reaction of 2,4-dinitrophenylhydrazine with pyruvate and/or oxaloacetate to yield a brown-colored complex in alkaline medium) were used. Serum ALP activity was evaluated using the spectrophotometric method. The results are expressed as units/liter (IU/L).

### 2.4. Histopathological Analysis

The livers were fixed in 10% neutral buffered formalin, sliced transversely, paraffin-embedded, and prepared as 5 *μ*m thick sections that were then stained with hematoxylin and eosin (H&E) for light microscopic evaluation. Three factors such as hepatocellular necrosis, level of inflammatory in portal area, and lymphocytic inflammatory infiltrations were evaluated using semiquantitative method described by Frei et al. The level of damage was recorded based on (0–4) grades in which grade 0 = no damage, 1 = very low level of damage, 2 = mild damage, 3 = moderate damage, and 4 = severe damage. Slides were viewed and photographed using a camera microscope (Labomed, LX400) at 400x magnification in at least three random microscopic fields from each animal by two expert pathologists without knowledge of the treatment groups.

### 2.5. Statistical Analysis

Data are presented as the mean ± SD. One-way analysis of variance (ANOVA) and Tukey's honestly significant difference (HSD) test were used for multiple comparisons of data. A *P* value less than 0.05 was considered to be significant. All measurements were replicated three times.

## 3. Results

### 3.1. Serum Toxicity Marker Enzymes

The effects of pretreatment with various dosages of the melatonin extract on elevation of the serum ALT, AST, and ALP activities following CP injection are shown in Table 1. Serum ALT, AST, and ALP activities were increased in all mice injected with CP relative to the untreated control animals (*P* < 0.001). Pretreatment with melatonin at all doses for 7 consecutive days mitigated CP toxicity and was associated with decreasing serum ALT, AST, and ALP activities. The serum ALT, AST, and ALP activities of the CP groups were 123.78 ± 4.24, 96.18 ± 2.07, and 185.86 ± 5.37 IU/L, while the control groups had significant lower serum ALT, AST, and ALP activities of 42.37 ± 1.46, 31.39 ± 1.48, and 52.28 ± 1.29 IU/L, respectively (*P* < 0.001). The maximum reduction in serum ALT, AST, and ALP activities was observed in mice that were pretreated with 20 mg/kg melatonin, which significantly decreased the serum ALT, AST, and ALP activities to 42.18 ± 2.09, 31.19 ± 2.28, 54.38 ± 4.91, and 31.19 ± 2.28 IU/L compared with CP-treated group, respectively, (*P* < 0.001). The data clearly show that melatonin has a suppressive effect on CP-induced hepatotoxicity in mice. The results indicate that pretreatment of mice with melatonin prevented serum ALT, AST, and ALP activities elevation following administration of CP in a dose-dependent manner.

### 3.2. Histopathological Examination of the Liver

**Figure 1:**
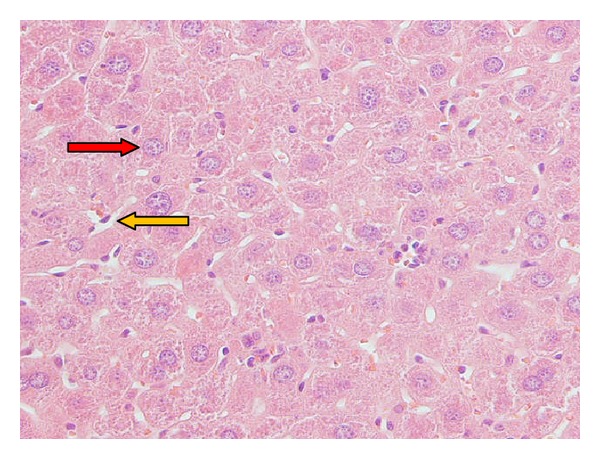
Normal group, a section in the liver of a mouse showing normal sinusoidal space (yellow arrow) and normal hepatocyte with polygonal shape (red arrow) (hematoxylin- and eosin-stained paraffin sections; H&E ×400).

**Figure 2:**
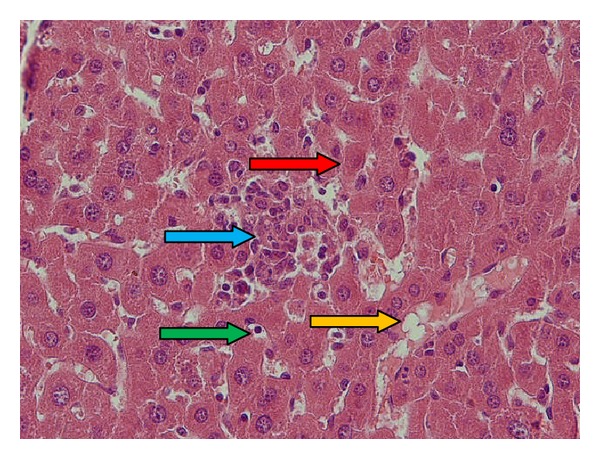
CP (200 mg/kg) group, a section of the liver of a mouse showing dilated and congested sinusoidal space (yellow arrow), lymphocyte between hepatocytes (green arrow), small portal space with moderate-to-severe inflammation (blue arrow), and necrotic small hepatocyte (red arrow) (hematoxylin- and eosin-stained paraffin sections; H&E ×400).

**Figure 3:**
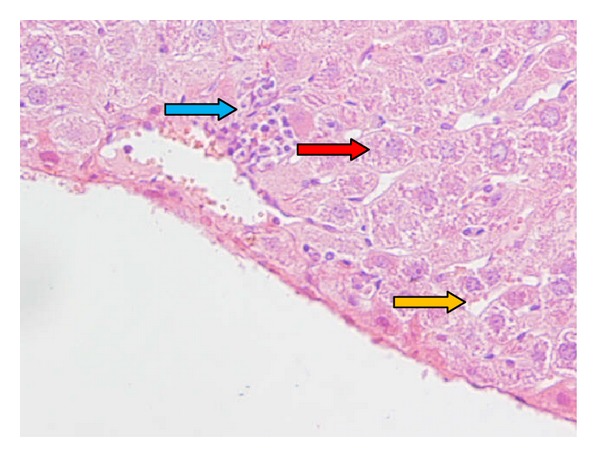
Pretreated animals with 20 mg/kg of melatonin for 7 days before CP administration; section in the liver of a mouse pretreated with 20 mg/kg of melatonin along with CP showing normal hepatocyte (red arrow), semicongested sinusoidal space (yellow arrow), and portal space with mild-to-moderate inflammation (blue arrow) (hematoxylin- and eosin-stained paraffin sections; H&E ×400).

Light microscopy photomicrographs of representative histological sections of the liver 24 hours after CP administration (200 mg/kg) compared with administration of melatonin extract (20 mg/kg for 7 consecutive days) as the optimum dose before CP treatment are shown in Figures 1–3.  Figure 1 shows a section in the liver of control mice showing normal hepatocyte with polygonal shape and normal sinusoidal space.  Figure 2 shows section of the liver of a mouse treated with CP showing dilated and congested sinusoidal space, lymphocyte between hepatocytes, small portal space with moderate-to-severe inflammation, and necrotic small hepatocyte.  Figure 3 shows section in the liver of a mouse pretreated with 20 mg/kg of melatonin along with CP showing semicongested sinusoidal space, normal hepatocyte, and portal space with mild-to-moderate inflammation.

The results of semiquantitative histopathological examination of the liver are also shown in Table 2. Briefly, the grades of damage for all three factors were 0 and 4 for nontreated control animals and animals receiving a single dose of CP at 200 mg/kg, respectively, and this indicates severe damage of the liver tissues 24 hrs after CP administration. Pretreatment of mice with melatonin for 7 consecutive days shows reduction in the level of liver tissue damage in a dose-dependent manner. These results are in agreement with the results observed in serum toxicity marker enzymes.

## 4. Discussion

Cyclophosphamide is effective against a wide spectrum of malignancies, such as leukemia, lymphoma, breast, lung, prostate, and ovarian cancers [19]. The parent compound is inactive in vivo and in vitro and exerts its biological activities through metabolites, mainly phosphoramide mustard, by hepatic microsomal enzymes [20]. Normal tissues injury or damage is the major limitation of using CP, which gives rise to numerous side effects; CP treatment also results in the production of reactive oxygen species (ROS), which cause peroxidative damage to vital organs [21]. The cellular and tissues toxicity were observed in the increased therapeutic dose of CP. We previously reported CP-induced oxidative stress and genotoxicity in mice bone marrow cells [22, 23]. We previously reported that CP increased the TBARS levels and therefore the extent of lipid peroxidation in the testis tissue of the experimental animals [24]. The cellular mechanisms by which CP causes liver injury are poorly understood; however, numerous studies have shown that CP treatment is associated with induction of oxidative stress by the generation of free radicals and ROS [25, 26]. We recently reported that herbal medicine containing high amount of natural products and antioxidant compounds ameliorated CP-induced oxidative stress and vital organs' toxicities. Thus, antioxidant biological compounds may help protect cells and tissues from the deleterious effects of CP-induced ROS and other free radicals [27–30].

Melatonin is principally synthesized by the pineal gland of mammals and has been suggested to have antioxidant and prophylactic properties. Melatonin scavenges a variety of reactive oxygen and nitrogen species including hydroxyl radical, hydrogen peroxide, singlet oxygen, nitric oxide, and peroxynitrite anion [31]. In our previous study, melatonin had dose-dependent inhibitor effects on CP-induced lipid peroxidation in mouse testicular tissue [24]. We further showed that melatonin had a potent genoprotective effect in preventing DNA damage induced by diazinon in human blood lymphocyte cells, and this protective effect may result from its free radical-scavenging properties [16]. Hence, we evaluated the protective effects of melatonin against hepatic damage induced by CP. Administration of melatonin for 7 consecutive days before CP injection showed a significant inhibition in the liver injury compared to CP-treated mice. Recently, we evaluated the protective effects of melatonin against CP-induced oxidative lung toxicity in mice. Administration of melatonin for 7 consecutive days before CP injection significantly inhibited the levels of lung lipid peroxidation compared to CP-only-treated mice. This activity could be due to the ability of melatonin to scavenge the free radicals generated by CP-induced oxidative stress where melatonin efficiently inhibited lipid peroxidation [32].

In the present study, the administration of CP damages the liver, and this observation is consistent with previous reports [33, 34]. Tissue damage due to CP might be alleviated due to the antioxidant property, free radical scavenging, increased activity of antioxidant defense system, and membrane stabilizing property of melatonin. CP administration under different conditions and doses has been demonstrated to be an excellent model to produce syndromes of both oxidative stress and hepatic damage [6, 35, 36]. In the present study, the activities of AST, ALT, and ALP are increased in the serum which is considered a marker of liver damage. Hepatic dysfunction was the most common regimen-related toxicity reported in patients treated with CP and total body irradiation [37]. Hepatic tissues were the primary sites for the microsomal activation of the drugs. Hepatic activation of CP leading to the formation of toxic metabolite caused damage to liver tissues as shown by increased liver enzymes in serum. The restoration of the levels of these marker enzymes in those animals intoxicated with CP and pretreated with melatonin indicates the protective activity of melatonin towards livers. This might be due to scavenging activity of melatonin against the toxic metabolite that was produced during the activation of the CP by liver microsomal enzymes. In the present study a single dose of CP administration (200 mg/kg body weight) to mice resulted in a significant increase in the activities of serum ALT, AST, and ALP. Pretreatment with melatonin also significantly lowered the levels of serum toxicity enzymes, and the values were comparable with that of the control animals. This suggests the hepatoprotective role of melatonin.

Histopathological studies proved that CP causes damage to the liver, and this was evidenced by the induced dilated and congested sinusoidal space, lymphocyte between hepatocytes, portal space with moderate-to-severe inflammation, and necrotic hepatocyte. This might be due to membrane damaging potential of the CP's metabolites. These pathological changes correlated well with the altered enzyme activities; these findings are compatible with other previous studies [38]. Pretreatment with melatonin effectively alleviated CP-induced histopathological changes in the liver and abnormal pathological findings of tissue injury and necrosis were reduced and tissues were protected from oxidative damage. The histopathological observations suggested the possibility of the melatonin being able to protect the tissues and thus decreasing the leakage of the enzymes (AST, ALT, and ALP) into the circulation.

## 5. Conclusion

The results support the protective role of melatonin against CP-induced hepatic damage. Melatonin also attenuated the activities of serum toxicity enzymes ALT, AST, and ALP. Histopathological examinations also confirmed the protective efficacy of melatonin against liver toxicity of CP. Therefore, melatonin may reduce the hepatotoxicity and liver damage induced by CP in mice through its ability to scavenge the ROS that induce lipid peroxidation and peroxidative damage and quench free radicals. Therefore, melatonin could be a potent candidate to use concomitantly as a supplement agent against hepatotoxicity of CP for the patients undergoing chemotherapy.

## Figures and Tables

**Table 1:** Effects of pretreatment with different doses of melatonin on serum toxicity marker enzymes along with CP administration.

Groups	Marker enzymes^a^		
	Serum AST (IU/L)	Serum ALT (IU/L)	Serum ALP (IU/L)
Control	42.37 ± 1.46	31.39 ± 1.48	52.28 ± 1.29
CP	123.78 ± 4.24^b^	96.18 ± 2.07^b^	185.86 ± 5.37^b^
Melatonin 2.5 mg/kg + CP	112.67 ± 3.38^c^	67.61 ± 1.19^f^	121.97 ± 0.35^c^
Melatonin 5 mg/kg + CP	73.54 ± 2.48^d^	54.32 ± 2.17^d^	87.29 ± 2.45^d^
Melatonin 10 mg/kg + CP	63.27 ± 2.61^d^	32.47 ± 2.61^e^	61.05 ± 3.31^d^
Melatonin 20 mg/kg + CP	42.18 ± 2.09^e^	31.19 ± 2.28^e^	54.38 ± 4.91^d^

CP: cyclophosphamide; ALP: alkaline phosphatase; ALT: alanine transaminase; AST: aspartate transaminase.

^a^Values are the mean ± standard deviation for each group of 5 mice. ^b^ *P* < 0.001 compared to the control; ^c^no significant difference compared to the control group; ^d^ *P* < 0.01 compared with the CP-treated group; ^e^ *P* < 0.001 compared with the CP-treated group; ^f^ *P* < 0.05 compared with the CP-treated group.

The data were analyzed with one-way ANOVA and Tukey's HSD test.

**Table 2:** The results of semiquantitative histopathological examination of the liver. Protection by melatonin at different doses against CP-induced tissue damage.

Factors/grades	Groups					
	Control	CP 200 mg/kg	Melatonin 2.5 mg/kg + CP	Melatonin 5 mg/kg + CP	Melatonin 10 mg/kg + CP	Melatonin 20 mg/kg + CP
Inflammatory and hemorrhage in portal area						
Grade 0	+					
Grade 1						+
Grade 2						
Grade 3			+	+	+	
Grade 4		+				
Hepatocellular necrosis						
Grade 0	+					
Grade 1						+
Grade 2			+	+	+	
Grade 3		+				
Grade 4						
Lymphocytic inflammatory infiltrations						
Grade 0	+					
Grade 1						+
Grade 2					+	
Grade 3			+	+		
Grade 4		+				
